# Deep Tissue Massage and Nonsteroidal Anti-Inflammatory Drugs for Low Back Pain: A Prospective Randomized Trial

**DOI:** 10.1155/2014/287597

**Published:** 2014-02-23

**Authors:** Marian Majchrzycki, Piotr Kocur, Tomasz Kotwicki

**Affiliations:** ^1^Department of Rheumatology and Rehabilitation, Poznan University of Medical Sciences, 28 Czerwca 1956 roku 135/147, 61-545 Poznan, Poland; ^2^Department of Kinesiotherapy, University School of Physical Education in Poznan, Królowej Jadwigi 27/39, 61-871 Poznan, Poland; ^3^Department of Pediatric Orthopedics and Traumatology, Poznan University of Medical Sciences, 28 Czerwca 1956 roku 135/147, 61-545 Poznan, Poland

## Abstract

*Objective*. To investigate whether chronic low back pain therapy with deep tissue massage (DTM) gives similar results to combined therapy consisting of DTM and non-steroid anti-inflammatory drugs (NSAID). *Design*. Prospective controlled randomized single blinded trial. *Settings*. Ambulatory care of rehabilitation. *Participants*. 59 patients, age 51.8 ± 9.0 years, with chronic low back pain. *Interventions*. 2 weeks of DTM in the treatment group (TG) versus 2 weeks of DTM combined with NSAID in the control group (CG). *Main Outcome Measures*. Visual analogue scale, Oswestry disability index (ODI), and Roland-Morris questionnaire (RM). *Results*. In both the TG and the CG, a significant pain reduction and function improvement were observed. VAS decreased from 58.3 ± 18.2 to 42.2 ± 21.1 (TG) and from 51.8 ± 18.8 to 30.6 ± 21.9 (CG). RM value decreased from 9.8 ± 5.1 to 6.4 ± 4.4 (TG), and from 9.3 ± 5.5 to 6.1 ± 4.6 (CG). ODI value decreased from 29.2 ± 17.3 to 21.4 ± 15.1 (TG) and from 21.4 ± 9.4 to 16.6 ± 9.4 (CG). All pre-post-treatment differences were significant; however, there was no significant difference between the TG and the CG. *Conclusion*. DTM had a positive effect on reducing pain in patients with chronic low back pain. Concurrent use of DTM and NSAID contributed to low back pain reduction in a similar degree that the DTM did.

## 1. Introduction

Treatment of patients with low back pain is based primarily on rehabilitation, which includes physical exercises and manual procedures, as well as pharmacotherapy. A small percentage of patients is treated surgically. Pharmacotherapy utilizes mainly nonsteroidal anti-inflammatory drugs, but in cases where severe pain occurs, opioid analgesics are used to amplify the treatment, and when increased muscle tone appears, the myorelaxant drugs are given [1–3].

In the treatment of chronic lumbosacral pain, therapy is aimed at improving patient's physical ability, relaxing the contracted structures and strengthening relevant muscle groups. Correct daily habits are taught, so incorrect and traumatizing body positions can be avoided. Additionally, the rehabilitation should instill the habit of daily physical activity [4].

Using nonsteroidal anti-inflammatory drugs to treat chronic pain in the lumbosacral area gives short term benefits; however, their use is poorly supported by the evidence [5, 6]. Many patients demonstrate side effects of nonsteroidal anti-inflammatory drugs [7–9]. At the same time, a limited number of papers point to the effectiveness of deep tissue massage.

We hypothesized that the use of therapy based on a series of 10 sessions of 30 minutes each consisting of deep tissue massage for chronic low back pain would have the same effect as the use of deep tissue massage combined with nonsteroidal anti-inflammatory drugs. Positive verification of the hypothesis could show the deep tissue massage to be effective and might result in limiting the overuse of nonsteroidal anti-inflammatory drugs in chronic low back pain patients.

## 2. Methods

Study subjects were recruited among patients admitted to the orthopedic and rehabilitation outpatient clinic.

The study was conducted on the group of 59 people (mean age: 51.8 ± 9.0 years). Recruitment was carried out among patients referred by physicians specialized in rehabilitation, orthopedics and traumatology, neurology, internal medicine, or rheumatology. The patients were referred to an ambulatory rehabilitation clinic with prescription to undergo a procedure of therapeutic massage. All study subjects suffered from low back pain, and they were diagnosed by a consultant with one of following: M47 (degenerative changes of the spine), M51 (other intervertebral discs diseases), and M54 (spine pain), according to the ICD-10 (International Classification of Diseases).

The patients with low back pain were classified as lumbosacral pain syndrome using one of the most popular classification tests—Quebec Task Force test [10–13]. The duration of the pain was classified as follows: up to 7 days, from 7 days to 7 weeks, and over 7 weeks.

The study used the criteria of inclusion and exclusion reported in Table 1.

**Figure 1:**
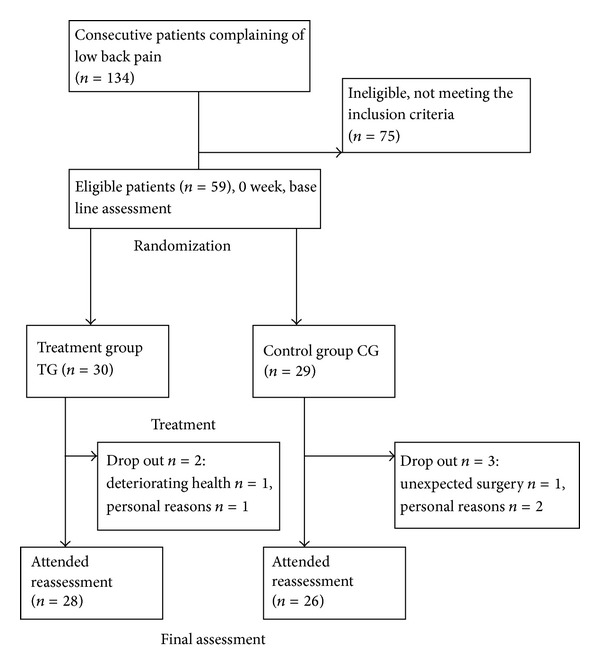
Flow of the participants through the trial.

Participants were randomly assigned in a 1 : 1 ratio to receive usual care, deep tissue massage—Treatment Group (TG)—or deep tissue massage and NSAID—Control Group (CG). The procedure of randomization was carried out using unmarked envelopes. The deep tissue massage was performed by certified therapists who did not know which patient belongs to which group. The flow of the participants through the trial is shown in Figure 1.

The Institutional Review Board of the Poznan University of Medical Sciences approved the study, resolution number 817/07. Each patient signed an informed consent form.

### 2.1. Interventions

Patients from both the treatment group and the control group underwent daily 30-minute session of deep tissue massage for 2 weeks (total of 10 sessions). In the control group, an additional pain relief was used in the form of nonsteroidal anti-inflammatory drugs. The drugs were used for symptomatic benefit but not more often than once daily.

### 2.2. Outcome Measures

The following functional questionnaires and pain scales were used: The Roland-Morris questionnaire (RM), the Oswestry disability index (ODI) [13, 14] and the visual analogue scale (VAS) [15]. The VAS was used three times in order to assess: (1) the pain intensity during resting (VAS1), (2) the pain intensity during motion (VAS2), and (3) the pain intensity during mobility of the aching area of the spine (VAS3).

The tests were performed twice: (1) before therapy, during the initial medical examination, and (2) one day after the therapy ended. The physician who performed the tests did not know which patient belonged to which group.

### 2.3. Statistical Analysis

The data distribution was assessed with the Shapiro-Wilk test. As data was not normally distributed, the comparisons between the two groups were assessed with Mann-Whitney test. For paired variables, the Wilcoxon test was used. *P* value of <0.05 was considered significant.

## 3. Results

Patients from the TG and the CG did not differ in basic characteristics before therapy (Table 2).

The results presented in Table 3 show that, in patients from both groups (TG and CG), the pain measured with the visual analogue scale was significantly reduced. The level of disability assessed with the use of the Roland-Morris Questionnaire and the Oswestry Disability Index revealed significant improvement compared to the baseline for both groups.

No statistically significant differences were observed between the groups with regard to baseline and end results as well as with regard to differences between the initial and final results.

## 4. Discussion

After having conducted MEDLINE, AMED, and Science Citation Index search, it was demonstrated that the functional questionnaires were most commonly used to measure the effect of back pain on daily activity of the patient [14]. Due to their methodological value and easiness in use, these tests are currently used in many countries as a basic research tool in people with low back pain. The questionnaires are used for patients with pain in the lumbar spine, but also with patients who experience pain radiating to the lower extremities [16, 17].

The study showed a significant improvement with regard to the experienced pain and the self-reported disability, both in the TG (deep tissue massage) and in the CG (deep tissue massage together with nonsteroidal anti-inflammatory drugs), which may suggest the effectiveness of deep tissue massage in back pain.

The Quebec Task Force test confirmed the validity of choosing the patients with chronic pain, and it excluded patients who underwent spine surgery up to 6 months before the study. The test also showed that most patients experienced pain without radiation and with no neurological symptoms. Those patients were susceptible to deep tissue massage therapy.

DTM is a form of massage used with “the understanding of the layers of the body and the ability to work with tissues in layers to relax, extend, and unlock the persisting, incorrect tensions, in the most effective and energy-efficient manner” [16]. Therapists working with this type of massage aim to change the soft tissues structure and limit the motion of the muscles. The knowledge of anatomy of locomotor system and the understanding of layer structure of tissues including fascia and muscles are needed. The therapist affects the tissues gradually until they respond with relaxation. Patient's body is put in proper positions, that is, muscles in the extended position. The therapist affects the muscle belly as well as the tendon-to-bone attachment, trying to soften the tendon and to influence receptors of muscle extension (Golgi organs of tendons) [16].

Medical literature contains very few studies showing that massage reduces lumbosacral pain in the acute stage. It was suggested, on the other hand, that massage in the subacute stage and in the early chronic stage of lumbosacral pain reduces the intensity and the quality of pain as effectively as a placebo therapy. Comparing the therapeutic effect of massage with other forms of therapy in lumbosacral pain, the results were similar to the effect of exercises and manipulation [18]. Research on patients with chronic lumbosacral pain suggested that massage was effective in reducing the intensity of pain and in improving patient's functionality. However, massage was not as effective in pain reduction as transcutaneous electrical nerve stimulation. On the other hand, authors showed that massage was more effective than relaxation, acupuncture, and mere health education [19]. Studies along with the research on patients with the visual analogue scale suggest that massage is effective in subacute stages and in chronic stages of unknown etiology (nonspecific pain). It is particularly effective when used along with exercises and when it is performed by an experienced therapist. The effectiveness of massage depends on the duration and the number of sessions, the surface area that undergoes massage, the strength of compression, and the patient's stress level [20]. Massage was reported to be more effective than placebo, patient education, acupuncture, muscle relaxation, and exercises increasing ability [21]. However, the assessment of massage effectiveness performed with functional questionnaires, the Roland-Morris Questionnaire and the Oswestry Disability Index, showed a small improvement in the functionality of patients with low back pain, and these results were not statistically significant. A small number of papers on deep tissue massage shows effectiveness of this form of massage in the treatment of myalgia symptoms [22], lowered systolic and diastolic blood pressure [23], and stronger effectiveness of deep tissue massage in comparison to therapeutic massage with regard to patient's pain sensation [24].

It is suggested that there are no individual and objective forms of therapy in chronic low back pain and that the spine care community needs to develop or update high-quality treatment guidelines [25]. Sole assessment of pain intensity is a difficult task and it is most often based on a subjective self-evaluation of the patient. The simultaneous use of different assessment scales helped to achieve more objective results.

The effectiveness of using NSAID in the treatment of chronic low back pain is questionable [26–28]. Some papers suggest a small improvement in wellbeing and a reduced subjective pain sensation in patients using certain NSAIDs when compared with the control group (placebo) [29, 30]. At the same time, there are no reports which would show that these drugs cause a long-lasting improvement in wellbeing or reduced subjective pain sensation [31]. Limited effectiveness of percutaneously absorbed nonsteroidal anti-inflammatory was reported [32]. Additionally, some studies suggest that patients have a dismissive attitude towards the drugs and do not believe in its effectiveness [26].

This study is one of the first studies assessing the effect of deep tissue massage on chronic lumbar pain. An attempt was made to check the effect of deep tissue massage on the possible reduction of nonsteroidal anti-inflammatory drugs. In our study we propose that the use of deep tissue massage causes fast therapeutic results and that, in practice, it could help to reduce the use of NSAID in the treatment of chronic low back pain.

### 4.1. Study Limitations

The eligibility of patients for the study was based on subjective criteria. Methods were based on patient's subjective experience of pain. In the future, the study protocol could be supplemented with objective functional tests, daily activity tests, and assessment of tissue tension.

This study did not distinguish between different nonsteroidal anti-inflammatory drugs. In the future similar studies could be conducted to evaluate the effectiveness of the therapy depending on the type of administered drug.

## 5. Conclusion

Deep tissue massage had positive effect on reducing pain in patients with chronic low back pain.

## Figures and Tables

**Table 1:** Criteria for inclusion or exclusion.

Inclusion in the study	Exclusion from the study
Age range: 40–60 years old	Age range: <40 years old or age >60 years old
Pain lasting longer than 7 weeks (chronic)	Acute pain
VAS1 ≥ 25 mm of 100 mm	VAS1 < 25 mm of 100 mm
VAS2 ≥ 25 mm of 100 mm	VAS2 < 25 mm of 100 mm
Lack of excluding factors mentioned in the right column of the table	Injection of local anesthetic Patients after surgical procedures around spine or in the abdominal area Neurological signs present Compression of spinal nerve root confirmed by specific imaging techniques: computer tomography, myelography, or magnetic resonance imaging Other diagnostic techniques, for example, electromyography, venography Diagnosis of (i) metastasis, (ii) vertebral fractures, (iii) spondylolisthesis, (iv) ankylosing spondylitis, (v) increased temperature (fever), (vi) pregnancy, (vii) inflammatory and acute ailments
	Nonsteroid anti-inflammatory therapy during the last 3 months or strong analgesic therapy (opioid and stronger)
	Allergy to ingredients of nonsteroid anti-inflammatory drugs
Informed consent of the patient to take part in the study	Lack of informed consent of the patient to take part in the study

**Table 2:** Basic characteristics of the groups.

	Treatment group (*N* = 28)		Control group (*N* = 26)		Significance of difference
Age	52.6 ± 7.4		50.8 ± 8.2		NS *P* = 0.40
Gender	Male *n* = 15		Male *n* = 13		NS *P* = 0.79
	Female *n* = 13		Female *n* = 13	
Duration of pain (weeks)	10.8 ± 2.4		11.9 ± 3.9		NS *P* = 0.22
Deep tissue massage applied	All		All		NA
Nonsteroidal anti-inflammatory drugs applied	None		All		NA
Number of DTM procedures	10		10		NA

Comparison of the groups based on classification of low-back disorders according to the Quebec Task Force on spinal disorders					

	Treatment group (*N* = 28)		Control group (*N* = 26)		Significance of difference
	Number	Percent	Number	Percent	

(1) Pain without radiation	28	100	26	100	*P*—NS for all comparisons between the treatment group and the control group
(2) Pain with radiation to lower limb proximally	3	12	4	15	
(3) Pain with radiation to lower limb distally	0	0	0	0	
(4) Pain with radiation to lower limb and neurological signs	0	0	0	0	
(5) Presumptive compression of a spinal nerve root on a simple radiogram, that is, spinal instability or fracture	0	0	0	0	
(6) Compression of a spinal nerve root confirmed by specific imaging techniques (computerized tomography, myelography, or magnetic resonance imaging) other diagnostic techniques, for example, electromyography and venography	0	0	0	0	
(7) Spinal stenosis	4	14	2	8	
(8) Postsurgical status, 1–6 weeks after intervention	0	0	0	0	
(9) Postsurgical status, >6 weeks after intervention (9.1) Asymptomatic (9.2) Symptomatic	0	0	0	0	
(10) Chronic pain syndrome	28	100	26	100	
(11) Other diagnoses	0	0	0	0	

NA: nonapplicable.

**Table 3:** Results of the visual analogue scale (VAS), Roland-Morris questionnaire (RM), and Oswestry disability index (ODI) before treatment (baseline test value) and after treatment (end test value).

Test	Group	Baseline test value	End test value	Wilcoxon test significance level	Difference value
VAS1	Treatment	58.3 [18.2]	42.2 [21.1]	∗	16.1
	Control	51.8 [18.8]	30.6 [21.9]	∗	21.2

VAS2	Treatment	56.1 [19.0]	36.5 [20.6]	∗	19.6
	Control	55.9 [16.6]	31.2 [21.2]	∗	24.7

VAS3	Treatment	47.4 [23.2]	33.5 [21.9]	∗	13.9
	Control	41.8 [21]	25.3 [19.4]	∗	16.5

RM	Treatment	9.8 [5.1]	6.4 [4.4]	∗	3.4
	Control	9.3 [5.5]	6.1 [4.6]	∗	3.2

ODI	Treatment	29.2 [17.0]	21.0 [15.1]	∗	8.2
	Control	21.4 [9.4]	16.6 [9.4]	∗	4.8

The values are given as mean with standard deviation in square brackets.

*Significance at *P* < 0.001.

VAS1—the pain intensity during resting; VAS2—the pain intensity during motion; VAS3—the pain intensity during mobility of the aching area of the spine.

## Abbreviations

DTM: Deep tissue massage
ODI: Oswestry disability index
RM: Roland-Morris Questionnaire
VAS: Visual analogue scale
VAS1: Visual analogue scale used in order to assess the pain intensity during resting
VAS2: Visual analogue scale used in order to assess the pain intensity during motion
VAS3: Visual analogue scale used in order to assess the pain intensity during mobility of the aching area of the spine
TG: Treatment group
CG: Control group
NSAID: Nonsteroidal anti-inflammatory drugs.
